# Current Status of PSMA-Radiotracers for Prostate Cancer: Data Analysis of Prospective Trials Listed on ClinicalTrials.gov

**DOI:** 10.3390/ph13010012

**Published:** 2020-01-13

**Authors:** Claus Zippel, Sarah C. Ronski, Sabine Bohnet-Joschko, Frederik L. Giesel, Klaus Kopka

**Affiliations:** 1 Faculty of Management and Economics, Witten/Herdecke University, 58448 Witten, Germany; Sabine.Bohnet-Joschko@uni-wh.de; 2 Praxis für Nuklearmedizin, Klinikum der Stadt Ludwigshafen, 67063 Ludwigshafen am Rhein, Germany; Sarah.Ronski@gmx.net; 3 Department of Nuclear Medicine, Heidelberg University Hospital, 69120 Heidelberg, Germany; Frederik.Giesel@med.uni-heidelberg.de; 4 Cooperation Unit Nuclear Medicine, German Cancer Research Center (DKFZ), 69120 Heidelberg, Germany; 5 Helmholtz-Zentrum Dresden-Rossendorf (HZDR), Institute of Radiopharmaceutical Cancer Research, 01328 Dresden, Germany; 6 German Cancer Consortium (DKTK), German Cancer Research Center (DKFZ), 69120 Heidelberg, Germany

**Keywords:** prostate cancer, PSMA tracer, registry data analysis, ClinicalTrials.gov, theranostics

## Abstract

The recent development of dedicated prostate-specific membrane antigen (PSMA) targeted radioligands shows the potential to change and improve the diagnosis and therapy of prostate cancer. There is an increasing number of prospective trials to further establish these tracers in the clinical setting. We analyzed data from the ClinicalTrials.gov registry including all listed prospective trials with PSMA-ligands for prostate cancer as of October 2019 concerning the different tracers and study characteristics. We found *n* = 104 eligible studies with a total of *n* = 25 different tracers in use: most frequently [^68^Ga]Ga-PSMA-11 (32%), followed by [^18^F]DCFPyL (24%) and [^177^Lu]Lu-PSMA-617 (10%). 85% are single-center, 15% multi-center studies. 95% national and 5% international studies. 34% are phase-II, 24% phase-I, 13% phase-I/-II, 12% phase-II/-III and phase-III and 7% early-phase-I. The primary purpose was classified as diagnostic in 72% of cases and therapeutic in 23% of cases. Most studies were executed in the USA (70%), followed by Canada (13%) and France (6%). This quantitative descriptive registry analysis indicates the rapid and global clinical developments and current status of PSMA-radioligands with emphasis on radiopharmaceutical and organizational aspects. It will be very interesting to see which tracers will prevail in the clinical setting.

## 1. Introduction

With the fairly recent invention of small molecule radiotracers targeting the prostate-specific membrane antigen (PSMA), a whole new field of nuclear medicine called theranostics started to arise [1,2,3,4]. These radioligands recently evolved from the preclinical into the clinical setting for the non-invasive (Hybrid-) PET imaging and radioligand-therapy of prostate cancer. Now, there is an ever increasing number of PSMA-tracers, in particular ^68^Ga-PSMA-, ^177^Lu-PSMA- or ^18^F-PSMA-radioligands, with very promising first results in retrospective clinical trials [5,6,7,8,9,10,11,12]. These studies show significant advantages for PSMA-radioligands in comparison to other nuclear medicine diagnostic methods like ^11^C- or ^18^F-Cholin-PET for the diagnosis, patient management and treatment of prostate cancer patients [13,14,15,16,17,18].

**Figure 1:**
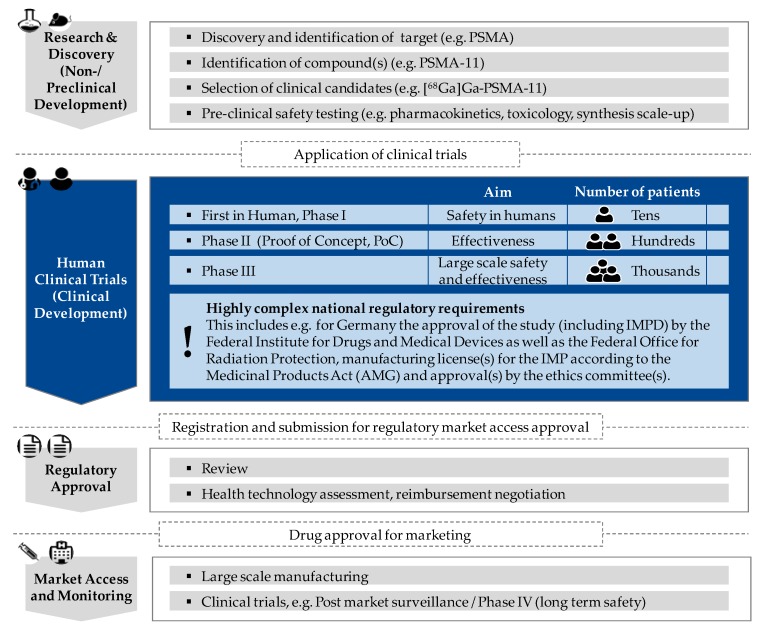
Schematic visualization of essential phases to be passed in the research and approval process of newly developed prostate-specific membrane antigen (PSMA)-radiopharmaceuticals/PSMA-radioligands with characteristics according to clinical trial phase; focus of the present study (clinical trial phase) in “blue”.

To ensure the continuing clinical development and use of these radioligands, there is an urgent need for prospective trials. In order for these promising radiopharmaceuticals to be applied in the routine clinical setting and find their way into the guidelines, the PSMA-tracers need to be approved by the respective authorities. To comply with the requirements, there is an increasing number of initiated prospective clinical trials of different phases to further advance PSMA-radioligands [19,20,21]. Figure 1 schematically shows the different phases of the admission process of innovative (radio-) pharmaceuticals in analogy to the admission process of all drugs and medicinal products. Clinical trials of this kind are paramount to ensure and validate the safety, efficacy and optimum dose of the drug to be applied. The initiation of prospective (multi-center) trials in nuclear medicine is especially complex because there are not only the Good Manufacturing Practice (GMP) guidelines for the production of not yet approved (radiopharmaceutical) medicinal products for clinical trials to follow but also laws and regulations of radiation protection to abide by and a plurality of permissions/licenses to obtain (e.g., study approval including investigational medicinal product dossier (IMPD), manufacturing license for the investigational medicinal product (IMP) and ethics approval).

### Research Question

With the quantitative analysis of data from the ClinicalTrials.gov registry, we aim to give an overview of the development and current status of prospective trials for PSMA-tracers in prostate cancer with emphasis on the organizational and radiopharmaceutical aspects including patient recruitment and study design. This systematic, data-based approach will enable us to appraise the current status of the translation of PSMA-radioligands from the preclinical to the clinical setting.

## 2. Material and Methods

### 2.1. Data Source

For our study, we used data provided by ClinicalTrials.gov, a free of charge, open access internet portal by the U.S. American National Institutes of Health for the registration of clinical trials (https://clinicaltrials.gov) [22]. This methodical approach has been used before, for example, in an oncologic context [23,24,25]. The platform is online since the year 2000. Its primary aim is to give structured information on world-wide initiated clinical trials, ongoing as well as completed, in one registry. This increases the transparency in the field of medical research and development. The registry is open to the public and provides an overview of the current status of clinical trials in a certain field [26,27]. ClinicalTrials.gov is a comprehensive platform collecting data on all kinds of (non-)interventional clinical studies including all types of study design, methodic approach, initiator/funder type, disease and/or medical product/drug. The data stems from trial specific entries by the principal investigator or responsible clinical research organization. After user registration and log-in, there is a standardized form to fill out including free-text sections, amongst others for the title and a short summary of the study as well as standardized drop-down sections concerning the study characteristics [28,29]. The latter include, amongst others, the study type and phase as well as organizational information like initiation date, recruitment status and trial site location(s).

### 2.2. Data Acquisition and Preprocessing

**Figure 2:**
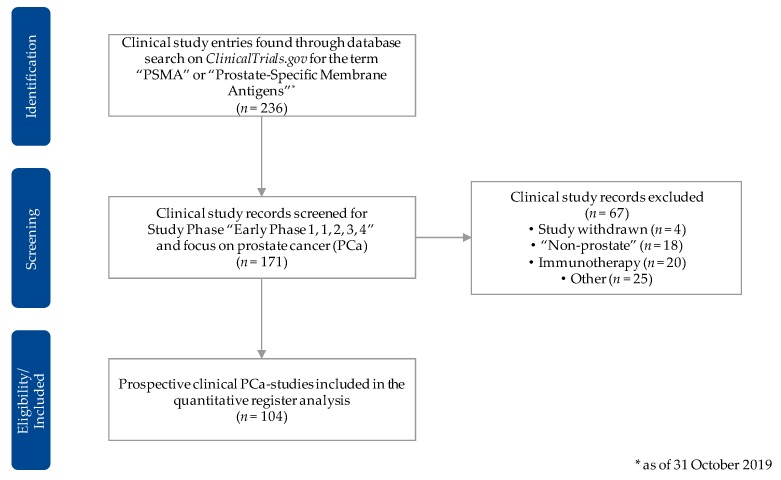
Flowchart for the selection procedure of the clinical study entries considered for the quantitative registry analysis. Source: Own figure based on the evaluation of the ClincalTrials.gov dataset [30].

For the data acquisition, we first of all used the “advanced search” option on the ClinicalTrials.gov website and scanned for all clinical trials with the key words “prostate-specific membrane antigens” and/or the acronym “PSMA.” We then exported the resulting data set, with the cutoff date being 31 October 2019, in the csv-format. Subsequently, we excluded all studies that were not categorized in one of the clinical phases (early phase I–phase IV) of a prospective clinical trial. We then screened the data set in detail to only include trials that used PSMA-radioligands for the diagnosis and/or treatment of prostate cancer. All trials that were categorized as “withdrawn” or did not primarily focus on PSMA-radiotracers for prostate cancer were excluded. We used further available information, including the free-text sections, to filter the trials accordingly and identify the applied radiotracer(s). Figure 2 shows the methodic approach to sustain the data set for our registry data analysis in a flowchart.

### 2.3. Evaluation Criteria

To give a general overview of the clinical development of prospective clinical trials concerning PSMA-radioligands for prostate cancer over the last couple of years, we sorted the trials according to their registration date (in years) and clinical trial phase. Furthermore, we processed information on study-specific parameters of three major categories, as seen below:

- Organization/cooperation: for example, study-specific collaborations and funding details;
- Recruitment: for example, the estimated enrollment, recruitment status and location;
- Study type/-design: for example, primary purpose of the study, clinical phase and type of masking or intervention model.

We then analyzed the different, specific PSMA-radiotracer(s) that were used and categorized the trials into either diagnostic or therapeutic approach [1,2]. There was no further standardized evaluation of details found in the free-text sections like intervention arms, inclusion criteria or end-points. 

### 2.4. Statistical Analysis

According to the explorative nature of our study’s aim, we conducted a descriptive analysis of the preprocessed data set. We determined the incidence of every criterion (absolute and relative value) and in case of ordinal-scale variables we also calculated mean, median, mode and standard deviation. To illustrate the development of prospective clinical trials over time, according to study phase, we used a bar plot. For the depiction of all other evaluated parameters we used tables. The recording, preprocessing and statistical analysis of our quantitative data set was done in Microsoft Excel^®^ for Microsoft Windows^®^.

## 3. Results

**Figure 3:**
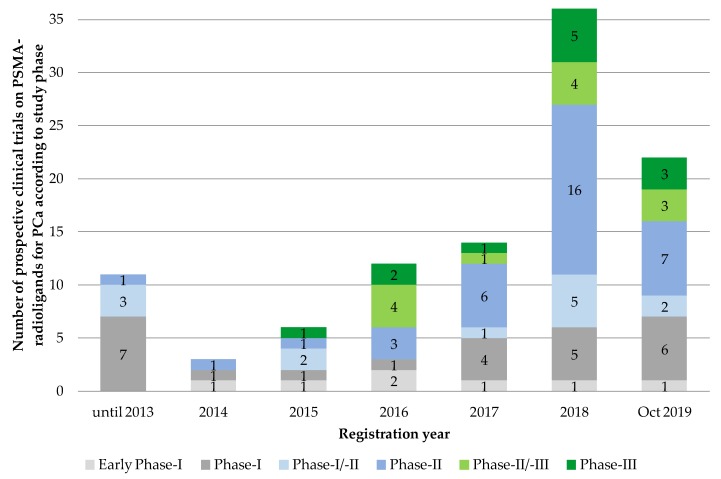
Number of prospective clinical trials on PSMA-radioligands for prostate cancer according to registration date and study phase (*n* = 104). Source: Own figure based on the evaluation of the ClincalTrials.gov dataset [30].

We found *n* = 104 eligible prospective clinical trial entries on PSMA-radioligands for the diagnosis and therapy of prostate cancer. Sorted by registration year, we found a continuous rise in the number of listed prospective PSMA trials from 2014 till 2018 with the steepest increase between 2017 and 2018, from *n* = 14 to *n* = 36 initiated trials. In 2019, this trend seems to curb with *n* = 22 newly registered trials at cut-off date 31 October 2019, although the validity of this statement is limited due to the (still) comparatively small number of clinical PSMA-trials analyzed. Due to the small numbers of registered trials per year before 2014, we decided to add up all listed trials before that year (Figure 3).

### 3.1. Applied PSMA-Radioligand 

In our study set, there was a total of *n* = 25 different PSMA-radiotracers in use, most commonly [^68^Ga]Ga-PSMA-11 (32%), [^18^F]DCFPyL (24%) and [^177^Lu]Lu-PSMA-617 (10%) (see Table 1). All of the remaining *n* = 22 PSMA-ligands, for example [^99m^Tc]Tc-MIP-1404, [^18^F]DCFBC or [^18^F]PSMA-1007 were applied less often, in a total of 35% of trials.

72% of trials were categorized as diagnostic, 23% as therapeutic, a minority of 3% as screening and 1% each as basic science and others.

### 3.2. Study Organization 

85% of all clinical trials in our data set were single-center, 15% multi-center studies. 95% were national and 5% international (see Table 1). The majority of trials (78%) was funded by individuals, universities or organizations themselves, 30% of trials were (co-) funded by the industry and 20% had a public sponsorship. In our study, the average clinical trial had 1.3 funding sources (SD ± 0.5).

### 3.3. Patient Recruitment 

With 58%, more than half of the listed trials were enrolling/recruiting patients at the time of data collection. 24% of trials were already completed. The estimated enrollment was 1–25 patients in 28% of trials, 26–50 patients in 22% of trials, followed by 101–250 patients in 20% of trials, 51–100 patients in 15% of trials, 251–1000 patients in 13% and more than 1000 patients in 1% of cases. On average, 145 patients were enrolled per trial (SD ± 231) with a median of 51 and a mode of 30. The vast majority of registered trials were conducted in the USA (70%), followed by Canada (13%), France (6%), Belgium (5%) and Australia (3%) (see Table 2).

### 3.4. Study Type and Design

Most PSMA-trials were listed as phase-II (34%), followed by phase-I (24%), phase-I/-II (13%), phase-II/-III and phase-III (12% each) with early phase-I (7%) in the rear (see Table 3). 

Regarding the group allocation of patients, 67% of the eligible trials did not specify a modus operandi, 16% each listed a randomized-controlled or a non-randomized design. 95% were open label/non-masked and 5% were masked. In more than two-thirds of cases, the group assignment was single-armed (69%), 19% were parallel and 6% each were sequential or cross-over assignments.

## 4. Discussion and Conclusions

The prostate-specific membrane antigen is a transmembranous glycoprotein with enzymatic value (synonym: glutamatcarboxypeptidase II) that is over-expressed in prostate cancer but not, as the name suggests, entirely specific for the prostate. It can also be found in healthy human tissue, for example, in salivary and lacrimal glands, the small intestine and the kidneys, which is of importance when using PSMA as a target for diagnostic and especially radioligand therapy [2]. PSMA-targeting radioligands, i.e., Glu-ureido based PSMA inhibitors bind to the zinc active site of PSMA. The inhibitor/PSMA complex becomes subsequently internalized into PSMA-positive tumor cells by clathrin-mediated endocytosis. This results in deposition of the PSMA-tracer both on the cell surface and in the cytosol where the tracer remains. At the same time, unbound tracer clears from the body. This leads to a high tumor-to-background ratio, making this class of theranostic radioligands feasible for sensitive imaging and efficient endoradiotherapy of PSMA-positive prostate cancer. As several retrospective studies show, the over-expression of PSMA in prostate cancer correlates directly and conveniently with the tumor grade, progression state and presence of metastases, and is a significant indicator for disease outcome (please see in all detail corresponding reviews on PSMA-tracers in [1,31,32,33]). 

This descriptive study gives an overview of the recent developments and the status quo of prospective clinical trials in the field of PSMA-radioligands for the diagnosis and therapy in prostate cancer patients. The strong increase of prospective clinical PSMA-trials registered on ClinicalTrials.gov shows how promising the use of PSMA radioligands has become in recent years for radiopharmaceutical and nuclear medical research and development. Our results can serve as a basis for radiopharmacists and nuclear medicine physicians as well as regulators and policy makers.

In order to give a detailed and informed description of the current status of PSMA-tracers, we carefully processed the original data set to glean and then to evaluate information concerning radiopharmaceutical and clinical parameters. Moreover, we point out specific characteristics and outline (regulatory) features which are special for prospective trials with innovative radiotracers for theranostics. In the subsequent sections, we will discuss the results of our registry data analysis in the context of the current literature from a radiopharmaceutical and organizational perspective. We will then delineate potential future fields of research and point out limitations of our analysis.

### 4.1. Development and Current Status of the Clinical Translation of PSMA-Radioligands

The clinical impact of PSMA-tracers for the diagnosis and therapy of prostate cancer in nuclear medicine can only further develop by initiation of (inter-)national prospective clinical trials. Only with these corresponding clinical trials can the safety and tolerability be confirmed, which is needed even when applying the microdosing concept. Moreover, of utmost interest is the determination of key variables such as sensitivity and specificity, and of the primary endpoints such as overall survival (OS) and progression free survival (PFS). With the establishment of these facts, the benefit for the patients can be proven unequivocally which, subsequently, will lead to newly implemented applications in nuclear medicine under marketing authorization.

We detected a fairly sudden and fast increase of prospective trials in the field of PSMA-tracers for prostate cancer, especially over the last years. Even though the concept of PSMA-targeting agents has been around for a long time [2], it was not till the development of small molecule PSMA-radiotracers and with it the new concept of theranostics, that PSMA-radioligands gained clinical relevance on a large scale. As our data illustrates, there is nowadays a vast number of different PSMA-radiotracers. Our study results show though, that, to date, in two-thirds (66%) of the investigated registered prospective trials, three PSMA-radioligands were by far the most frequently validated. As may be deduced from Table 1, these are tracers with different radionuclides (^68^Ga vs. ^18^F for diagnostic purposes and ^177^Lu for radioligand-therapy). It will be very interesting to see which tracers will prevail in the long run. It is to be presumed that only a very small number of PSMA-tracers with unique characteristics, and/or application fields that show favorable radiopharmaceutical and clinical properties, will prevail in the clinical setting. In this context, it has to be noted that the tracers that we found to be the most frequently applied in prospective clinical trials as of now, will not necessarily be the ones to prevail in the future, especially since our analysis did not include any kind of evaluation or assessment of the radiopharmaceutical and clinical abilities and performance of the different tracers.

Beyond that, our study results show that there is a high number of PSMA-tracers coupled with ^68^Ga and ^18^F respectively. Taking into consideration the different radiopharmaceutical and clinical properties as well as assets and draw-backs concerning production, transport and handling of the radioligands, this seems very sensitive. For the production of ^68^Ga-PSMA-radioligands for example, a ^68^Ge/^68^Ga-generator is primarily used whereas you need a highly regulated cyclotron infrastructure for the production of ^18^F-tracers. Advantages of the latter include a higher production yield and a longer half-life compared to ^68^Ga-ligands (110 vs. 68 min). It is, therefore, possible to transport ^18^F-tracers over a certain distance, for instance, to external hospitals or application sites for the (Hybrid-) PET scan [34]. Taking into account these specific differences in the manufacturing and transportation process, there is also an economic incentive for the use of one or the other radionuclide. Hospitals with a rather small number of prostate cancer patients and/or without a cyclotron on-site could opt for ^68^Ga-PSMA-radioligands produced with a ^68^Ge/^68^Ga-generator, because the acquisition of a cyclotron comes with a great financial and organizational investment. Clinics, with a high number of patients needed to treat, and a cyclotron on-site, on the other hand, will generally pick the ^18^F-radioligands as their PSMA-tracer of choice amongst others because of the decreasing marginal costs. As an alternative, they could also opt for the production of ^68^Ga-tracers via the cyclotron if there is only a need for a small amount for in-house use.

Tracers for radioligand-therapy enable the personalized treatment of prostate cancer patients applying the theranostic principle and, therefore, cover a different application spectrum. There is a total of *n* = 18 trials in our data set that we identified by means of the reported applied tracer as well as the study description as therapeutic. With *n* = 15 ^177^Lu was, by far, the most frequently used radionuclide for radioligand therapy. With *n* = 2 for ^131^I and *n* = 1 ^225^Ac, the alternatives were scarce. ^177^Lu is currently one of the favorable beta particle emitting radionuclides for endoradiotherapy. Its production is possible with research reactors which are also used for the production of ^131^I and ^99^Mo. Its nuclear decay properties make ^177^Lu feasible and optimal for interval short-term applications. Due to the small amount of gamma emission, the radioligand distribution can be monitored by scintigraphy and/or SPECT. Additionally, with ^177^Lu there are less side effects because beta irradiation on salivary and lacrimal glands results mainly in reversible xerostomia.

### 4.2. Study Organization and Patient Recruitment

Taking a closer look at the organizational structures of the included clinical trials, it has to be noted that the vast majority were single-center studies who recruited their patients at one trial site only. The main reason for this could be the considerably smaller organizational and financial effort of monocentric trials in comparison to decentralized recruitment at different trial sites. In order to conduct prospective multicenter trials with PSMA-radioligands, there are a huge number of requirements to meet or to agree upon, i.e., approvals like positive ethics votum and a production license for each trial site, highly regulated guidelines to follow including laws from different federal and local authorities (including radiation protection), the harmonized production and application of the IMP, calibrated PET-cameras for the scans at the different sites and, last but not least, the documentation and storage of left-over trial tracers. Especially in the case of prospective multicenter trials for PSMA-radioligands with a short half-life like ^68^Ga-tracers, there is the difficulty of producing the PSMA-tracers harmonized and in accordance with the legal and regulatory guidelines, as well as being GMP-compliant at all the different trial sites. Zippel, Neels et al. are discussing the most relevant aspects of initiating a prospective multicenter trial with short-lived PSMA-radioligands by means of one of the trials included in our data set ([^68^Ga]Ga-PSMA-11 in high-risk Prostate Cancer, NCT03362359) for the D-A-CH region [35]. 

The data analysis further shows that with 95% of all trials, the vast majority were national studies. Only 5% of all trials recruited patients internationally. One reason could be a strategic edge in the approval process. Probably equally important are the difficulties obtained by differing regulatory requirements for the production of the IMP and trial admission in different countries [36]. The VISION-Trial, a study about radioligand-therapy with [^177^Lu]Lu-PSMA-617, was the only multicenter trial in the data set that is recruiting patients in different countries and across continents (NCT03511664) [37,38].

The authors strongly believe that the initiation of (inter-/national) multicenter trials will gain importance in the very near future, in order to achieve the needed number of patients to be enrolled for prospective clinical trials for radiotracers in a foreseeable time frame and within the limits of narrow inclusion criteria in a more and more individualized treatment regimen in precision oncology. Keeping in mind the aforementioned regulatory and organizational challenges, especially where short-lived tracers like ^68^Ga are concerned, it seems sensible to further strengthen already existing regulatory set-ups for the decentralized manufacturing and application of PSMA-radiotracers with sufficient recruitment numbers to facilitate a quick and therewith comparatively cheap translation of promising new radioligands into the clinic. This applies specifically for investigator-initiated trials, which make up the vast majority in our data set, with usually limited financial resources and/or a tight budget.

### 4.3. Research Perspectives

The rapid increase in registered prospective PSMA-trials on ClinicalTrials.gov over the last couple of years (see Figure 1) is a clear indicator for the increasing importance of the collection of patient-based endpoints and proof of patient benefit derived from prospective trials in nuclear medicine. This applies to diagnostic as well as therapeutic PSMA-tracers. This descriptive study may only give a first indication and point out a trend regarding the recent developments of prospective PSMA-trials for prostate cancer. The authors state that further research in the field is needed, especially in the areas of but not necessarily limited to:

- Multivariate analyses, for example of comparable study designs;
- Study (design) specific subanalyses of the different PSMA-radiotracers and their application fields (primary/secondary staging, BCR, radioligand-therapy, patient management);
- Compare and combine the prostate cancer-related trial registry entries on ClinicalTrials.gov with the data from other registries such as from Europe (e.g., DRKS), Asia (JPRN, ChiCTR) or Oceania (ANZCTR);
- Longitudinal studies to identify future developments in the field of novel PSMA-radiotracers, be they diagnostic or therapeutic;
- Additional detailed analyses of the free-text sections of the evaluated registry data concerning, for example, primary/secondary end points, inclusion/exclusion criteria and outcome measures.

Furthermore, there was a number of trials concerning PSMA-radiotracer-use for non-prostate cancer in the original, not processed data set; for example, in thyroid cancer, different kinds of gynaecological cancers, renal cancer, transitional cell carcinoma, glioblastoma and other solid tumors. We excluded these trials because they did not fit our study’s aim. The assessment of potential benefits and perspectives in this field are, therefore, beyond the scope of our analysis. Further research in this direction could be of great value.

### 4.4. Limitations

The registry data from ClinicalTrials.gov enables analyses of a multitude of systematically collected, study-specific detailed information of high quality over a period of time. A method-inherent error of our investigative approach is, that our data set only represents a subset of all initiated PSMA-trials around the globe, since the PI or sponsor of the respective clinical trial may as well choose a different registry to list their study accordingly. Examples, as listed above, are the Deutsches Register für Klinische Studien (DRKS) in Germany or the Australian New Zealand Clinical Trials Registry (ANZCTR). The latter includes, for instance, the so called “LuPSMA trial” a phase-II trial for radioligand-therapy with [177Lu]Lu-PSMA-617 (Identifier: 12615000912583) [20], that is listed exclusively at ANZCTR. It is possible and, as our data confirms, quite common to register a trial in more than one registry, which again makes a comparison of data between different registries quite difficult due to potential duplications. In addition, different (usually national) study registries collect trial data with differing reporting options and obligations, and have different study search functions and data export functions resulting in a very heterogeneous information set when compared to other registries. This illustrates the importance of harmonizing the fairly large number of registries to prospectively create (also linguistically) more uniform data sets. This, in turn, would facilitate and improve the knowledge transfer from a methodological point of view.

Furthermore, common limitations of clinical registry (meta-) data analyses apply, which may impair the data quality [23,24,25,39]. This includes, in particular, incorrect or not at all answered sections of the registry form. While processing our data set, we detected, for example, that multiple studies stated “treatment” as a primary study purpose even though the PSMA-tracers applied were clearly for diagnostic purposes. Sometimes this was the case, when the trial used PSMA-tracers to decide on how to proceed with patient treatment or how the imaging affects the treatment or radiation plan. 

Last but not least, it has to be assumed that since ClinicalTrials.gov is an U.S. American registry, there is a disproportionately high number of registered clinical trials conducted in North America. Our study results strongly support this hypothesis (see Table 2), seeing that the vast majority of trials recruited in the USA and Canada. This may possibly lead to distortions in comparison to the status of PSMA-tracer-trials in, for example, Europe or Asia. Our study’s aim was, therefore, solely to illustrate a trend in this recently evolving field of nuclear medicine. Additionally, since ClinicalTrials.gov is by far the biggest and most renowned registry for clinical trials with currently more than 320,000 listed clinical trials from all over the world [40], the authors conclude that this is a very suitable data set for an overview on the current status of PSMA-radioligands. 

## Figures and Tables

**Table 1:** Applied radiotracers and organizational parameters of the included prospective clinical prostate cancer-related trials with PSMA-radioligands from the ClinicalTrials.gov registry (*n* = 104).

	Absolute (*n*)	Relative (%) *
**Applied PSMA radioligand**		
[^68^Ga]Ga-PSMA-11	33	32
[^18^F]DCFPyL	25	24
[^177^Lu]Lu-PSMA-617	10	10
Other (e.g., [^99m^Tc]Tc-MIP-1404, [^18^F]DCFBC, [^18^F]PSMA-1007)	36	35
**Organization/Cooperation**		
***Single-center/Multi-center***		
Single-center	88	85
Multi-center	16	15
***National/International***		
National	99	95
International	5	5
Funding source **		
Industry	31	30
NIH	20	19
Other U.S. Federal agency	1	1
All others (individuals, universities, organizations)	81	78

* Sum partly ≠ 100 due to rounding. ** More than one choice possible. Source: Own table based on the evaluation of the ClinicalTrials.gov dataset [30].

**Table 2:** Recruitment parameters of the included clinical prospective prostate cancer-related trials with PSMA-radioligands from the ClinicalTrials.gov registry (*n* = 104).

	Absolute (*n*)	Relative (%) *
Study status **		
***Patient recruitment***		
Open	60	58
Not open	44	42
***Recruitment status***		
Recruiting	50	48
Completed	25	24
Active, not recruiting	13	13
Not yet recruiting	10	10
Enrolling by invitation	5	5
Unknown status	1	1
**Estimated enrollment (number of patients)**		
1–25	29	28
26–50	23	22
51–100	16	15
101–250	21	20
251–1000	14	13
>1000	1	1
Location/country ***		
United States of America	73	70
Canada	14	13
France	6	6
Belgium	5	5
Australia	3	3
Other (Austria, Germany, UK, China, etc.)	15	14

* Sum partly ≠ 100 due to rounding. ** Trials with status reported as “withdrawn” or “terminated” not included. *** More than one choice possible. Source: Own table based on the evaluation of the ClinicalTrials.gov dataset [30].

**Table 3:** Study type and study design specific parameters of the included clinical prospective trials with PSMA-radioligands for prostate cancer from the ClinicalTrials.gov registry (*n* = 104).

	Absolute (*n*)	Relative (%) *
**Study phase**		
Early Phase-I	7	7
Phase-I	25	24
Phase-I/-II	13	13
Phase-II	35	34
Phase-II/-III	12	12
Phase-III	12	12
**Primary purpose**		
Diagnostic	75	72
Treatment	24	23
Screening	3	3
Basic Science	1	1
Other	1	1
**Allocation**		
Non-Randomized	17	16
Randomized	17	16
Not specified	70	67
**Masking**		
None (Open Label)	99	95
Masked	5	5
*Single (investigator)*	*1*	*1*
*Single (participant)*	*2*	*2*
*Double or triple*	*2*	*2*
**Intervention model**		
Single Group Assignment	72	69
Parallel Assignment	20	19
Sequential Assignment	6	6
Crossover Assignment	6	6

* Sum partly ≠ 100 due to rounding. Source: Own table based on the evaluation of the ClinicalTrials.gov dataset [30].

## References

[1] Kopka K., Benesova M., Barinka C., Haberkorn U., Babich J. (2017). Glu-ureido-based inhibitors of prostate-specific membrane antigen: Lessons learned during the development of a novel class of low-molecular-weight theranostic radiotracers. J. Nucl. Med..

[2] Virgolini I., Decristoforo C., Haug A., Fanti S., Uprimny C. (2018). Current status of theranostics in prostate cancer. Eur. J. Nucl. Med. Mol. Imaging.

[3] Hope T.A., Afshar-Oromieh A., Eiber M., Emmett L., Fendler W.P., Lawhn-Heath C., Rowe S.P. (2018). Imaging prostate cancer with prostate-specific membrane antigen pet/ct and pet/mri: Current and future applications. AJR. Am. J. Roentgenol..

[4] Ruigrok E.A.M., van Weerden W.M., Nonnekens J., de Jong M. (2019). The future of psma-targeted radionuclide therapy: An overview of recent preclinical research. Pharmaceutics.

[5] Eder M., Schafer M., Bauder-Wust U., Hull W.E., Wangler C., Mier W., Haberkorn U., Eisenhut M. (2012). ^68^Ga-complex lipophilicity and the targeting property of a urea-based psma inhibitor for pet imaging. Bioconj. Chem..

[6] Eder M., Neels O., Muller M., Bauder-Wust U., Remde Y., Schafer M., Hennrich U., Eisenhut M., Afshar-Oromieh A., Haberkorn U. (2014). Novel preclinical and radiopharmaceutical aspects of [^68^Ga]Ga-PSMA-HBED-CC: A new pet tracer for imaging of prostate cancer. Pharmaceuticals.

[7] Benesova M., Schafer M., Bauder-Wust U., Afshar-Oromieh A., Kratochwil C., Mier W., Haberkorn U., Kopka K., Eder M. (2015). Preclinical evaluation of a tailor-made dota-conjugated psma inhibitor with optimized linker moiety for imaging and endoradiotherapy of prostate cancer. J. Nucl. Med..

[8] Cardinale J., Martin R., Remde Y., Schafer M., Hienzsch A., Hubner S., Zerges A.M., Marx H., Hesse R., Weber K. (2017). Procedures for the gmp-compliant production and quality control of [(18)f]psma-1007: A next generation radiofluorinated tracer for the detection of prostate cancer. Pharmaceuticals.

[9] Cardinale J., Schafer M., Benesova M., Bauder-Wust U., Leotta K., Eder M., Neels O.C., Haberkorn U., Giesel F.L., Kopka K. (2017). Preclinical evaluation of (18)f-psma-1007, a new prostate-specific membrane antigen ligand for prostate cancer imaging. J. Nucl. Med..

[10] Robu S., Schottelius M., Eiber M., Maurer T., Gschwend J., Schwaiger M., Wester H.J. (2017). Preclinical evaluation and first patient application of 99mtc-psma-i&s for spect imaging and radioguided surgery in prostate cancer. J. Nucl. Med..

[11] Chen Y., Pullambhatla M., Foss C.A., Byun Y., Nimmagadda S., Senthamizhchelvan S., Sgouros G., Mease R.C., Pomper M.G. (2011). 2-(3-{1-carboxy-5-[(6-[18f]fluoro-pyridine-3-carbonyl)-amino]-pentyl}-ureido)-pen tanedioic acid, [18f]dcfpyl, a psma-based pet imaging agent for prostate cancer. Clin. Cancer Res..

[12] Cho S.Y., Gage K.L., Mease R.C., Senthamizhchelvan S., Holt D.P., Jeffrey-Kwanisai A., Endres C.J., Dannals R.F., Sgouros G., Lodge M. (2012). Biodistribution, tumor detection, and radiation dosimetry of 18f-dcfbc, a low-molecular-weight inhibitor of prostate-specific membrane antigen, in patients with metastatic prostate cancer. J. Nucl. Med..

[13] Afshar-Oromieh A., Malcher A., Eder M., Eisenhut M., Linhart H.G., Hadaschik B.A., Holland-Letz T., Giesel F.L., Kratochwil C., Haufe S. (2013). Pet imaging with a [^68^Ga]gallium-labelled psma ligand for the diagnosis of prostate cancer: Biodistribution in humans and first evaluation of tumour lesions. Eur. J. Nucl. Med. Mol. Imaging.

[14] Afshar-Oromieh A., Babich J.W., Kratochwil C., Giesel F.L., Eisenhut M., Kopka K., Haberkorn U. (2016). The rise of psma ligands for diagnosis and therapy of prostate cancer. J. Nucl. Med..

[15] Giesel F.L., Hadaschik B., Cardinale J., Radtke J., Vinsensia M., Lehnert W., Kesch C., Tolstov Y., Singer S., Grabe N. (2017). F-18 labelled psma-1007: Biodistribution, radiation dosimetry and histopathological validation of tumor lesions in prostate cancer patients. Eur. J. Nucl. Med. Mol. Imaging.

[16] Rahbar K., Ahmadzadehfar H., Kratochwil C., Haberkorn U., Schafers M., Essler M., Baum R.P., Kulkarni H.R., Schmidt M., Drzezga A. (2017). German multicenter study investigating ^177^Lu-PSMA-617 radioligand therapy in advanced prostate cancer patients. J. Nucl. Med..

[17] Rowe S.P., Macura K.J., Mena E., Blackford A.L., Nadal R., Antonarakis E.S., Eisenberger M., Carducci M., Fan H., Dannals R.F. (2016). PSMA-based [^18^F]DCFPyL PET/CT is superior to conventional imaging for lesion detection in patients with metastatic prostate cancer. Mol. Imaging Biol..

[18] Rowe S.P., Macura K.J., Ciarallo A., Mena E., Blackford A., Nadal R., Antonarakis E.S., Eisenberger M.A., Carducci M.A., Ross A.E. (2016). Comparison of prostate-specific membrane antigen-based 18f-dcfbc pet/ct to conventional imaging modalities for detection of hormone-naive and castration-resistant metastatic prostate cancer. J. Nucl. Med..

[19] Nielsen J.B., Zacho H.D., Haberkorn U., Nielsen K.M., Dettmann K., Langkilde N.C., Petersen L.J. (2017). A comprehensive safety evaluation of ^68^Ga-labeled ligand prostate-specific membrane antigen 11 pet/ct in prostate cancer: The results of 2 prospective, multicenter trials. Clin. Nucl. Med..

[20] Hofman M.S., Violet J., Hicks R.J., Ferdinandus J., Thang S.P., Akhurst T., Iravani A., Kong G., Ravi Kumar A., Murphy D.G. (2018). [^177^Lu]-PSMA-617 radionuclide treatment in patients with metastatic castration-resistant prostate cancer (lupsma trial): A single-centre, single-arm, phase 2 study. Lancet Oncol..

[21] Fendler W.P., Calais J., Eiber M., Flavell R.R., Mishoe A., Feng F.Y., Nguyen H.G., Reiter R.E., Rettig M.B., Okamoto S. (2019). Assessment of ^68^Ga-PSMA-11 pet accuracy in localizing recurrent prostate cancer: A prospective single-arm clinical trial. JAMA Oncol..

[22] McCray A.T., Ide N.C. (2000). Design and implementation of a national clinical trials registry. J. Am. Med. Inform. Assoc. JAMIA.

[23] Hirsch B.R., Califf R.M., Cheng S.K., Tasneem A., Horton J., Chiswell K., Schulman K.A., Dilts D.M., Abernethy A.P. (2013). Characteristics of oncology clinical trials: Insights from a systematic analysis of ClinicalTrials.gov. JAMA Intern. Med..

[24] Bell S.A., Tudur Smith C. (2014). A comparison of interventional clinical trials in rare versus non-rare diseases: An analysis of ClinicalTrials.gov. Orphanet J. Rare Dis..

[25] Chen Y.P., Lv J.W., Liu X., Zhang Y., Guo Y., Lin A.H., Sun Y., Mao Y.P., Ma J. (2017). The landscape of clinical trials evaluating the theranostic role of pet imaging in oncology: Insights from an analysis of ClinicalTrials.gov database. Theranostics.

[26] Zarin D.A., Tse T., Ide N.C. (2005). Trial registration at ClinicalTrials.gov between may and october 2005. N. Engl. J. Med..

[27] Zarin D.A., Tse T., Williams R.J., Califf R.M., Ide N.C. (2011). The ClinicalTrials.gov results database—Update and key issues. N. Engl. J. Med..

[28] McCray A.T. (2000). Better access to information about clinical trials. Ann. Intern. Med..

[29] Zarin D.A., Keselman A. (2007). Registering a clinical trial in ClinicalTrials.gov. Chest.

[30] U.S. National Library of Medicine ClinicalTrials.gov → Advanced Search. https://clinicaltrials.gov/ct2/search/advanced.

[31] Kiess A.P., Banerjee S.R., Mease R.C., Rowe S.P., Rao A., Foss C.A., Chen Y., Yang X., Cho S.Y., Nimmagadda S. (2015). Prostate-specific membrane antigen as a target for cancer imaging and therapy. Q. J. Nucl. Med. Mol. Imaging.

[32] Rahbar K., Afshar-Oromieh A., Jadvar H., Ahmadzadehfar H. (2018). Psma theranostics: Current status and future directions. Mol. Imaging.

[33] Kratochwil C., Haberkorn U., Giesel F.L. (2019). Radionuclide therapy of metastatic prostate cancer. Semin. Nucl. Med..

[34] Kesch C., Kratochwil C., Mier W., Kopka K., Giesel F.L. (2017). ^68^Ga or ^18^F for prostate cancer imaging?. J. Nucl. Med..

[35] Zippel C., Neels O.C., Hennrich U., Giesel F.L., Kopka K. (2019). Initiation of clinical multicentre studies with local radiotracer production—Regulatory environment and radiopharmaceutical-organisational aspects. Nuklearmed. Nucl. Med..

[36] Decristoforo C., Penuelas I., Patt M., Todde S. (2017). European regulations for the introduction of novel radiopharmaceuticals in the clinical setting. Q. J. Nucl. Med. Mol. Imaging.

[37] Rahbar K., Bodei L., Morris M.J. (2019). Is the vision of radioligand therapy for prostate cancer becoming a reality? An overview of the phase iii vision trial and its importance for the future of theranostics. J. Nucl. Med..

[38] Iravani A., Violet J., Azad A., Hofman M.S. (2019). Lutetium-177 prostate-specific membrane antigen (PSMA) theranostics: Practical nuances and intricacies. Prostate Cancer Prostatic Dis..

[39] Califf R.M., Zarin D.A., Kramer J.M., Sherman R.E., Aberle L.H., Tasneem A. (2012). Characteristics of clinical trials registered in ClinicalTrials.gov, 2007–2010. JAMA.

[40] Zarin D.A., Tse T., Williams R.J., Carr S. (2016). Trial reporting in ClinicalTrials.gov—The Final Rule. N. Engl. J. Med..

